# Developing of Multidimensional Perspectives Checklist of Professionalism for Undergraduate Occupational Therapy Students in Assistive Technology Service: Delphi Study

**DOI:** 10.3390/ijerph19127028

**Published:** 2022-06-08

**Authors:** Chia-Hui Hung, Yu-Ming Wang, Cheng-Yi Huang, Chung-Hui Lin

**Affiliations:** 1 Department of Occupational Therapy, Chung Shan Medical University, Taichung 40201, Taiwan; chhung@csmu.edu.tw; 2 Department of Occupational Therapy, Chung Shan Medical University Hospital, Taichung 40201, Taiwan; 3 Department of Psychology, Chung Shan Medical University, Taichung 40201, Taiwan; wym@csmu.edu.tw; 4 Clinical Psychological Room, Chung Shan Medical University Hospital, Taichung 40201, Taiwan; 5 Department of Nursing, Chung Shan Medical University, Taichung 40201, Taiwan; huangcy@csmu.edu.tw; 6 Department of Nursing, Chung Shan Medical University Hospital, Taichung 40201, Taiwan

**Keywords:** professionalism, assistive technology, checklists, occupational therapy, Delphi survey, assessment

## Abstract

Professionalism is a critical attribute that occupational therapy students must establish throughout education, especially in the context of assistive technology (AT). This study aimed to construct a multidimensional perspectives checklist (MPC) from the stakeholders to reflect professionalism development in the AT service courses. Fifteen experts in AT clinical practice and educators (N = 15) consented to and completed three rounds of the Delphi survey. Data were qualitatively analyzed to form a preliminary checklist in the first round. In rounds two and three, participants rated their level of agreement with the MPC items. A descriptive analysis of consensus was performed. Thirty items were classified into five subscales (teachers, therapists, patients, peers, learners) and fit into a framework with three dimensions and seven categories. After the Delphi survey, the MPC achieved high consensus, convergence, and stability. Two additional categories of professionalism emerged in the study, namely aesthetic and personal characteristics and reflection. The MPC developed in this study reflects the perspectives of various stakeholders in occupational therapy practice, providing helpful information for students to prepare themselves. Therefore, the MPC could contribute to expanding and developing the scope of professionalism in occupational therapy, especially in AT service.

## 1. Introduction

Professionalism is a critical attribute that occupational therapy students must establish throughout their education. Professionalism is an evolving, socioculturally informed, multidimensional construct that encompasses behaviors and attributes expected of an occupational therapist by society [1]. Traditionally, professionalism was considered a competence that students would gain through spending time with role models in academic and clinical fields [2]. The definition of professionalism has recently come to include professional roles, professional identity, competencies, and responsibilities that support excellence in professional practice, leading to therapist-patient relationships, patient satisfaction, and healthcare outcomes [3]. The notion of professionalism in medicine has three essential domains: clinical competence, reflective capacity, and ethics [3,4,5]. However, the changing nature of the organizational and social milieu in which medicine operates creates a dynamic situation where no definition has been universally agreed upon as definitive [3,6]. Despite these complexities, education for professionalism in medical school is crucial in the 21st century [4,7].

Occupational therapy is a healthcare profession that engages clients in the therapeutic use of everyday life occupations to support their occupational performance and participation [8]. Professionalism in occupational therapy practice is a complex combination of competencies [9,10]. Several competencies have been identified so far, such as professional knowledge, critical thinking, clinical reasoning, quality service, patient- or client-centeredness, timeliness of service, collaboration, and teamwork, as well as respect for the profession, department, and company, and self-management skills [11,12,13,14]. However, as healthcare and societal needs change, opportunities and new areas of occupational therapy practice emerge; as a result, professional attributes are growing and shifting. New competencies are required for occupational therapy students for role-making and professional identities. They need to be prepared to meet the expectations of society [3,15].

Assistive technology (AT) is one of the areas of occupational therapy practice; however, in the recent decade, the service model has shifted from a biomedical model to a client-centered model [15]. The AT is defined as any item, piece of equipment, software program, or product system used to increase, maintain, or improve the functional capabilities of persons with disabilities [16]. Occupational therapists in AT services demonstrate a unique approach that considers the person from a client-centered perspective. Occupational therapists also integrate the knowledge of society, technology, engineering, art, and medicine to deliver AT interventions, including professional assessment, selection, provision, education, and training in using high- and low-tech assistive technology, applying universal design principles, and recommendations for changes to the environment or activity for the client’s ability to engage in daily occupations [8]. In recent years, with the advancement and convenience of technology and industrial technology, AT services delivery includes equipment and devices customized to be made for a single user or mass-produced for a larger population [17]. Therefore, occupational therapists must have more clinical and ethical attributes and competencies to care for clients directly. At the same time, they must also be technically and scientifically equipped to apply relevant technology, engineering, and aesthetics in order to make the AT for clients [4,16,17].

Professionalism is a multidimensional construct that may generate different needs when taking on different roles or intervention perspectives [3]. However, not only are there limited mentors and role models in the learning stage [4], but it is also not easy to understand the various needs of individual cases for AT services. For example, researchers found that when occupational therapy students were involved in providing AT services, they were more concerned with the overall design and making of the AT than with clients’ considerations [18,19]. Therefore, occupational therapy educators should guide students to understand professionalism in the AT service context. Thus, this paper proposes that students must understand the competency requirements of explicit precision in professionalism. Precision refers to the accuracy of the label and the associated competencies. Let students understand that these necessary items are the professional competencies required to become an occupational therapist of the AT service [4,20].

Professionalism has evolved from a core competency at the level of an individual characteristic to a broader interpretation at the social interaction level [21]. Professionalism is related to the nature and context of learning activities and is co-constructed through interaction with key stakeholders [22,23]. Therefore, when the professionalism assessment is viewed at the level of social interaction, meaningful collective input from all stakeholders in the clinical and learning context, such as the faculty staff, the clients, peer students, and teachers, is necessary [24]. Nonetheless, to the best of our knowledge, the necessary items of concern for these stakeholders remain unknown in recent studies. Therefore, it is imperative to create a precise checklist of the competencies to provide a framework to define requirements to keep students focused on professionalism development. Thus, this study aimed to develop a multidimensional perspectives checklist (MPC) of professionalism for undergraduate occupational therapy students enrolled in assistive technology service courses to assess their professionalism during their studies.

## 2. Materials and Methods

The Delphi survey is a group facilitation technique that aims to achieve consensus on the opinions regarding essential questions from experts through a series of structured questionnaires [25]. The Delphi process is cyclical, with panel members providing input and comments repeatedly and researchers feeding back summaries of opinions within the group while allowing for interaction and engagement among panel members [26]. This study used the Delphi survey as a research method and sought to attain consensus among an expert panel on the items of a professionalism checklist for students in an occupational therapy assistive technology design and practice program. Ethical approval was obtained from Chung Shan Medical University Hospital (project no. CS1-20130) for this study.

### 2.1. Participants

Assistive technology services are provided by a multiprofessional team, including physical therapists and occupational therapists, responsible for designing, producing, and managing interventions. The interdisciplinary input of physical therapists was considered when research involved developing an assistive technology curriculum or assessment tools [27]. Therefore, the current Delphi survey participants included an expert panel of occupational therapists and physical therapists to incorporate diverse opinions.

Presently, there is no agreement in the literature concerning expert panel size [28]. However, Duffield [29] suggests that when a Delphi panel is homogenous, 10 to 15 people are adequate. As previously mentioned, 15 experts were selected to participate in this study as an expert panel. Based on the purposive and criterion-based sampling method, criteria included: participants’ commitment to the research purpose, gaining AT service competency through experience or qualifications, and demonstrating interest in AT service and clinical teaching. They had a homogenous practical character and had working experience with stakeholders of the current research. Therefore, they could provide opinions on patient concerns from their experience of clinical therapeutic interaction. They could also offer an understanding of students’ clinical learning needs based on their teaching experience. Invitations were sent via e-mail, explaining the purpose of the study; we assured the participants of their anonymity.

Students and standardized patients (SP) were invited to participate in the Delphi study. Students who were undergraduate occupational therapy students taking AT-related courses were invited to participate. Standardized patients who had received SP training and had participated in AT simulation learning as a standardized patient and had scoring experience were considered to meet the inclusion criteria.

### 2.2. Procedure

The Delphi process was commenced in February 2021 and completed in June 2021. Three rounds of the Delphi survey were conducted through e-mail. Feedback on the results of the previous round was provided in each subsequent round [25,30]. All participants were invited to complete each Delphi round, unless they indicated they wanted to withdraw from the study. Reminder e-mails were sent to the participants at 7 and 14 days following the dissemination of each survey round.

The first-round survey aimed to generate a list of items that participants considered should be reported when describing the professionalism of an assistive technology provider. The initial survey comprised three sections, including a brief description of the study and overview of the Delphi process, demographic information, and one open-ended question. The unstructured, open-ended question asked the participants their opinions on what essential concepts should be considered when evaluating an assistive technology provider’s performance, especially from the perspective of multidimensional evaluators, including the teachers, therapists, clients, student peers, and the students themselves. An example from a researcher with some detail was provided to prompt participants to report their opinions. Consent was inferred by participants’ qualitative comments. The responses to the question asked in the first round were integrated into the researcher’s preliminary checklist items. At this stage, we conducted two further processes. First, we removed the duplicate items. Second, we made checklist items available in advance to standardized patients and students to confirm whether the content covered their needs and the description was easy to read and evaluate. These confirmed items were then formed in the first draft of the MPC and were quantitatively fed back to the participants through a second-round Delphi survey questionnaire. The second and third rounds of the Delphi survey aimed to fulfill the consensus process. In each round, participants received a summary of the results from the previous round, as well as instructions for completing the survey. This process was repeated until consensus was reached or until the number of returns for each round decreased.

The second Delphi survey included a summary of the round one results: the percentage of participants who mentioned the necessity of the items based on the qualitative inputs. Participants were invited to adequately assess each item’s relevance on a five-point Likert scale (5, highly relevant; 1, very irrelevant) and further comment on the questions. Participants were invited to indicate whether each item should be omitted, possibly kept, or whether they considered the item to be essential or desirable to the checklist. They could also provide additional rewording suggestions, comments, or questions for items that did not reach consensus in round 1 and new items proposed by participants during the first round [31].

After statistical analysis regarding the participants’ collective opinion, the third quantitative questionnaire was derived from the second questionnaire. The respondents used a five-point Likert scale (5, highly appropriate; 1, very inappropriate) to provide opinions about the appropriateness of each item’s content. The third Delphi survey informed the respondents about the current status of their collective opinion. It helped to rate the appropriateness of the final analysis items, which of them respondents may think unimportant, or their perceptions of the items’ compliance [27].

### 2.3. Data Analysis

In the first-round survey, each participant was allocated a random identification number for reporting and collecting the results. Demographic data were collated and summarized for the research group.

The responses to the first round of the survey were analyzed qualitatively. Due to the responses of experts having their context, therefore, the study used the textual analysis method as the analytic technique to analyze themes in the first-round survey responses [32]. The first author read the responses multiple times and opened coding throughout the responses to construct the coding scheme. Then, the second author used the coding scheme to code all the responses. Two researchers individually reviewed and summarized the data, eliminated redundancies, and coded and categorized the responses using an inductive approach. The kappa coefficient was calculated to provide a measure of interrater reliability [33]. Then, the researchers met to discuss the dimensions and concepts that they had identified using the ground theory [32]. The discussion was undertaken until agreement was reached. Data triangulation was achieved by comparing these findings with field notes, standardized patients’ and students’ comments, and literature reviews [34]. The researchers then met again to combine the concepts and consider the dimensions required to form the evaluation items of the MPC; the researchers collaborated to refine each item statement using the participants’ original wording as much as possible and complete the survey content for the second round.

The data produced in the second and third rounds were analyzed using the statistical package IBM SPSS 23. Descriptive statistics were computed for each item, including the mean, median (Mdn), standard deviation (SD), and interquartile range (IQR). The content validity ratio (CVR) was calculated to assess the level of consensus, using the equation CVR = (Ne − N/2)/(N/2), in which Ne is the number of panelists indicating “essential” (the two positive options on the scale) and N is the total number of panelists. The CVR values suggested that consensus was achieved when at least two-thirds of the participants strongly agreed with each item [35,36]. The coefficient of variation was computed as the SD divided by the mean to measure the stability of verification. The formula IQR/2 was used to determine the degree of convergence. The degree of consensus was calculated using 1 − (IQR/Mdn). When stability was 0.8 or less, convergence was 0.5 or less, and the consensus was 0.7 or more, the item was considered statistically significant and consensus was deemed to have been reached [37,38].

## 3. Results

### 3.1. Demographic Data

A total of 15 experts participated in the study (53.3% female, 46.7% male) with a mean age of 39.3 years old (range, 24–54). Most participants had a master’s degree (53.3%); 26.4% had a Ph.D. and 20% had a bachelor’s degree. All participants were also experienced in professional teaching and practice (average of 9.2 and 12.7 years of professional teaching experience and clinical experience, respectively). Most participants were occupational therapists (73.3%); four were physical therapists (26.7%). All 15 experts participated in the three rounds (response rate = 100%). Detailed characteristics of participants in the expert panel are summarized in Table 1.

Three standardized patients (mean age = 35.3) and twenty undergraduate occupational therapy students (mean age = 22.1) were also invited to participate in the content review process of the first round of the preliminary checklist. However, they did not participate in the second and third round of the survey.

### 3.2. Delphi Survey

The expert panelists volunteered 146 items in round one, with an average of 10 items per panelist (range from 5 to 27 items). No additional items were added after round three. After being reviewed by the standardized patients and students, 60 items were removed due to duplication of items, vague descriptions, and difficulty assessing the clinical learning situation. The remaining 86 items were categorized into the five subscales of the MPC. Percentage agreement and the interrater reliability of the two researchers were calculated for each subscale. The first round MPC had moderate agreement for the subscales of clients (k = 0.50, *p* = 0.008), peers (k = 0.48, *p* = 0.000), and the learners, students themselves (k = 0.58, *p* = 0.000), and almost perfect agreement for the subscales of teachers (k = 0.77, *p* = 0.000) and therapists (k = 0.86, *p* = 0.000). The percentage agreement between the two researchers was high for all subscales (73.3–95.7%; see Table 2).

Round two of the Delphi survey was designed to assess the relevance of the MPC using a five-point Likert scale for the 86 items identified in round one. After analyzing the response values, 36 items were deleted due to a CVR value less than 1, SD value larger than 1, mean less than 4, and IQR larger than 0.5. After the researchers met to consider the dimensions to form the checklist items of the MPC, a total of 50 items were obtained.

Round three was designed to check the appropriateness and consensus among the participants for these 50 items. Appropriateness was very high (*n* = 45, 90%) or high (*n* = 5, 10%) across all domains. No item was assessed as moderate or unimportant. The third-round survey had a higher average CVR, convergence, consensus, and stability than the second round (Table 3). Thus, all 50 items in the MPC were retained after round three. The 50 items belonged to five subscales (10 items per subscale) (Table 4); however, each subscale had several items that mention the same competence requirements as another subscale; therefore, three professionalism domains and seven categories were developed as a framework for integration. Finally, 30 items were reserved in the MPC (Table 5).

### 3.3. The Structure of MPC Items

The MPC items were classified into three dimensions of professionalism (skills for clinical competence, humanistic qualities, and reflective capacity) [3]. They were further subdivided into seven categories under the following domains: therapist-patient communication, personal characteristics and reflection, managing work, interpersonal/teamwork/society, clinical skills and demonstration, aesthetics, respect, patience, and empathy. Theme selection for domains and categories reference the American Occupational Therapy Association (AOTA) educational guidelines for competent occupational therapists [39]. The occupational therapist education standards provided by AOTA serve as a reference for developing a competent occupational therapist. However, occupational therapists require slightly different competencies in different healthcare areas. Precise guidelines should be provided when educating the AT providers, which is also the meaning of MPC. The method of theme selection is first to classify the items. One must carefully read the content of the items of the same category, extract the main concepts, and then select the appropriate concept from the AOTA educational standards to subsume the content of this category [32]. The final blueprint of the MPC is formed and presented in Table 4.

### 3.4. The Manual of MPC

Through the validation process of the Delphi method, the MPC was designed as a valid and structured instrument for developing, implementing, and evaluating professionalism associated with assistive technology service courses.

The MPC is composed of five subscales. Each represents a stakeholder in the learning context of the AT service. A subscale can examine the development of professionalism from a stakeholder’s point of view. The five subscales can be used independently or together in the same context. The ten items to which each subscale belongs are contained in the “Subscale” column of Table 5. Each item used to assess learner performance corresponds to a five-point Likert scale ranging from 1 (very poor) to 5 (excellent). As a result, the maximum rating for each subscale is 50, and the total score of MPC is 250. Table 6 is an example of teacher subscales.

The design concept of MPC came from the social nature of learning tasks and learning situations. Therefore, MPC is suitable as an assessment instrument for simulation learning. Course instructors can incorporate stakeholders such as therapists, standard patients, and peers to create a learning context to guide and facilitate learning. As examiners, stakeholders can use the MPC to provide learners with the most authentic feedback based on their interactive experience.

For learners, MPC provides adequate and structured guidelines for role-making. After the learner obtains the score from a stakeholder, he/she can understand his/her learning results from the stakeholder’s perspective. Learners can interactively compare the scores provided by stakeholders and refer to the blueprint to understand the overall effectiveness of their learning, therefore setting up the directions of preparation for becoming competent AT providers.

## 4. Discussion

This study aimed to develop an MPC for undergraduate occupational therapy students to reflect the development of professionalism associated with assistive technology service courses. The result of the Delphi survey yielded 30 items that were categorized into a framework with three dimensions and seven categories.

Over the past few decades, shifts in occupational therapy education to competency-based education have promoted the emergence of new, practical education programs and assessments [40,41]. Assistive technology provision is an emerging area of practice in occupational therapy [3]. In recent years, most of the research on AT has focused on developing assistive devices intervention and the provision model. However, little mention is made of assistive technology services’ training and learning effectiveness. Therefore, research in AT service is necessary to support the need for occupational therapy programs to reexamine the content of AT training [42,43]. The Taiwan Occupational Therapy Association [44] highlighted that career development is required for beginners and experts across clinical fields to improve competencies and professionalism. This study is the first to examine the professionalism development of undergraduate students in learning assistive technology services to provide a basis for further development of courses or programs.

The MPC generated by three rounds of a Delphi survey encompasses diverse views on the stakeholders, with dimensions of competence to construct the assessment. The framework of MPC includes three dimensions: skills for clinical competence, humanistic qualities, and reflective capacity, which are consistent with domains of professionalism covered in the literature [3]. The seven categories of MPC are consistent with the core competencies proposed by the Taiwan Occupational Therapy Association [44]; furthermore, the MPC includes two additional categories: aesthetics and personal characteristics and reflection. The former is related to being aware of the sensitivity and sensibility of external objects in occupational therapy practice, along with several concepts in the aesthetic dimension, including understanding the spirit of the AT service, innovation, creativity, and emphasizing aesthetic appeal. The latter is related to being aware of oneself [39], including professional attitude and identity, a sense of responsibility, and awareness and reflection of the quality of the learning task.

Aesthetics has the essence of experience. In occupational therapy, aesthetic experience plays an essential role in the acceptance and uptake of AT [45]. Aesthetic experience is one element that renders clients willing to let AT into their lives and makes life more meaningful [46]. However, the literature has found that aesthetics is often overlooked when providing occupational therapy services [47], and even users have negative comments about the aesthetics of the AT provided [45]. The scientific model approaches for clinical reasoning are well-documented and are frequently applied in the occupational therapy process [48,49,50], while the aesthetics of occupational therapy practice means the quality of the practice’s form, including the provision of ATs that meet the aesthetic appeal of individual cases. Such a reasoning process requires a deeper understanding and observation of the experience and needs of the case. It is narrative clinical reasoning, which includes empathizing with and respecting the feelings of the case when making clinical decisions [39]. Focus needs to be placed on aesthetic appeal while maintaining all the benefits of the AT when recommending interventions [45,51]. Therefore, clinicians and providers of occupational therapy education need to acknowledge the importance of aesthetics. However, how to use teaching strategies to improve the competence of aesthetics still requires further in-depth research.

The category of personal characteristics and reflection is traditionally considered to be expected of healthcare professionals when referring to professionalism. Robert et al. proposed that when professionalism is seen at the personal level, the measurement of behaviors, cognitive processes, and attitudes will be focused on [22]. While professionalism is regarded on an interpersonal level, equity, altruism, and communication skills can be incorporated into the dimension of personal characteristics and reflection as predictors of professionalism development [12].

In the field of assistive technology service, the categories of personal characteristics and reflection and aesthetics can be translated into the specific competency for work with patients, sensing patients’ needs, and the ability to reflect and carry out an aesthetic process to make an assistive technology. Therefore, a program of reflective and aesthetic education should be included in formal curricula to facilitate the development of professional attitudes, and better methods for examining educational outcomes and increasing the students’ professionalism should be developed.

The MPC developed in this study to reflect students’ professionalism includes the perspectives of various stakeholders in occupational therapy practice, including the teachers, therapists, patients, peers, and the learners themselves. Currently existing professionalism assessments are mainly self-assessments at the personal level, with limitations related to obtaining various perspectives in an evaluation situation. While AT service provision is in the context, therefore, the design and selection of the assessment of professionalism should be undertaken with a systematic approach, including personal, interpersonal interaction, and social levels as considerations [22]. Thus, an instrument that reflects professionalism from the perspectives of various stakeholders is required. The teachers’ and peers’ perspectives subscales of the MPC reflect academic learning, and the subscales of the therapists’ and patients’ perspectives reflect the students’ ability in clinical practice. Therefore, the MPC may be beneficial for guiding students studying assistive technology development and consulting the curriculum. The perspectives, content, and dimensions of the MPC guide students to achieve professionalism and align with the contract between healthcare professionals and society. Moreover, the MPC enables teachers to deliver more rewarding, effective, and individualized teaching. Therefore, this study represents a significant development, as it establishes a foundation for academic and clinical development of professionalism for assistive technology practice by enabling an assessment of various multidimensional perspectives of practice.

### Limitations and Further Research Directions

This study has some limitations. First, the generalization of this study is limited because we only focused on assistive technology courses. Therefore, additional studies are required to reflect students’ professionalism development in other health curricula. Second, the MPC developed in this study does not provide a norm of evaluation score because the norms for professionalism have not yet been completely defined. Therefore, it is necessary to establish the norms for different stages of learning so that the level of professionalism can be interpreted to examine the effectiveness of education programs. Third, the development of MPC is based on the consensus of experts through the Delphi survey, and it has expert validity. However, the psychometric properties of the checklist need to be provided in further research.

## 5. Conclusions

This study developed an MPC to reflect learning related to assistive technology development and consulting. Thirty items were classified into five subscales and were fit into a framework with three dimensions and seven categories through the Delphi survey. After three rounds, an MPC with high consensus, convergence, and stability was achieved for assessing professionalism in assistive technology development and consulting. The MPC reflects the perspectives of various stakeholders in occupational therapy practice. In addition, the MPC can provide helpful information as it establishes multidimensional measurements for therapist-patient communication, personal characteristics and reflection, managing work, interpersonal/teamwork/society, clinical skills and demonstration, and aesthetics. Quantitative feedback based on the MPC may facilitate reflection and the development of professionalism among students. Therefore, the MPC developed in this study could contribute to expansion and development of the academic and clinical scope of professionalism in occupational therapy.

## Figures and Tables

**Table 1:** Demographic data on the survey participants.

Characteristics	Participants (*N* = 15) *n* (%)
Respondents in Round (*n*)	
1 (*n* = 15)	15 (100)
2 (*n* = 15)	15 (100)
3 (*n* = 15)	15 (100)
Gender	
Female	8 (53.3)
Male	7 (46.7)
Professional discipline	
Occupational therapy	11 (73.3)
Physical therapy	4 (26.7)
Academic degree	
PhD	4 (26.7)
MSc	8 (53.3)
BSc	3 (20.0)
Length of practical experience in assistive technology	
10 years	9 (60.0)
5–10 years	3 (20.0)
<5 years	3 (20.0)
Length of teaching experience for assistive technology	
>10 years	8 (53.3)
5–10 years	1 (6.7)
<5 years	6 (40.0)

**Table 2:** Percentage agreement and kappa scores for the subscales of the MPC.

Subscales	% Agreement	Kappa	Strength of Agreement *
Teachers (*n* = 20)	90%	*k* = 0.77, *p* = 0.000	Almost perfect
Therapists (*n* = 23)	95.7%	*k* = 0.86, *p* = 0.000	Almost perfect
Clients (*n* = 12)	75%	*k* = 0.50, *p* = 0.008	Moderate
Peers (*n* = 15)	73.3%	*k* = 0.48, *p* = 0.000	Moderate
Learners (*n* = 18)	77.8%	*k* = 0.58, *p* = 0.000	Moderate

* The benchmarks for the strength of agreement between raters based on the kappa are rated as poor (k < 0.00), slight (k = 0.00–0.21), fair (k = 0.21–0.40), moderate (k = 0.41–0.60), substantial (k = 0.61–0.80), and almost perfect (k = 0.81–1.00) [31].

**Table 3:** Comparison of the results of the second and third rounds of the Delphi survey (N = 15).

Delphi Round	Mean	Median	SD	CVR	Kappa	Convergence	Consensus	Stability
Second	4.55	5	0.59	0.978	0.74	0.41	0.84	0.13
Third	4.71	5	0.48	0.982	0.98	0.25	0.9	0.10

SD: standard deviation; CVR: content validity ratio; Kappa: kappa coefficients.

**Table 4:** Blueprint of the MPC items: subscales, dimensions, and categories.

	Dimensions							
	Skills for Clinical Competence			Humanistic Qualities		Reflective Capacity		
Categories Subscales	TPC	ITS	CSD	A	RPE	PCR	MW	Total
Teachers	1	-	7	2	-	-	-	10
Therapists	1	1	7	-	-	1	-	10
Patients	2	-	6	-	1	-	1	10
Peers	-	4	2	1	-	2	1	10
Learners	1	1	3	1	-	3	1	10
Subtotal	5	6	25	4	1	6	3	50

Categories: TPC: therapist-patient communication; ITS: interpersonal/teamwork/society; CSD: clinical skills and demonstration; A: aesthetics; RPE: respect, patience, and empathy; PCR: personal characteristics and reflection; MW: managing work.

**Table 5:** Final items included on the MPC after the Delphi survey.

Domains of Professionalism	Categories	Item Statement	Subscales *
Skills for Clinical Competence	Therapist-patient communication	1. Communicate with patients and families in an easy- to-understand manner	1, 2, 3, 5
		2. Ability to interpret and explain interventions	3
	Clinical skills and demonstration	3. Demonstrate professional clinical reasoning	1
		4. Carefully assess symptoms and needs for assistive technology interventions	1, 2, 3, 4
		5. Understand the skills and details of assistive technology service	1
		6. Understand the concept, evaluation process, and intervention methods of assistive technology	1, 2, 5
		7.Understand basic body structure and biomechanics related to diseases and diagnosis	1, 2, 3, 5
		8. Ability and knowledge to develop organizational sequence for making assistive technology	1, 2, 4, 5
		9. Apply a designed assistive technology for patients, enable the patient to perform daily living activities in an easy and comfortable manner	2, 3
		10. Provide easy-to-understand health education for patients (including usage, side effects, prognosis)	1, 2, 3
		11. Use the whole-person approach to provide client-centered intervention	2
		12. Analyze the pros and cons of each treatment plan for patients	3
		13. Help patients to learn and adapt to the use of assistive technology	3
		14. Pay attention to “operational safety”	4
	Interpersonal/ teamwork/ society	15. Listen to multiprofessional comments	2
		16. Fully participate in teamwork	4
		17. Dyadic communication and provide advice (comments) at the right time	4
		18. Friendly cooperation and mutual support	4, 5
Humanistic Qualities	Respect, patience, and empathy	19. Have patience, respect, and empathy	3
	Aesthetics	20. Understanding the spirit of the AT service	1
		21. Innovation and creativity	1, 4
		22. Emphasize aesthetic appeal when making and designing assistive technology	5
Reflective Capacity	Personal characteristics and reflection	23. Professional identity, present a professional attitude	2
		24. Complete projects for which one is personally responsible conscientiously	4
		25. Share and deliver the correct message, respect and accept peers’ comments	4
		26. Self-directed learning to find relevant knowledge and skills	5
		27. Ability to review and revise the learning process, then prepare for subsequent study	5
		28. Aware of the appearance and functions of end-product of assistive technology	5
	Managing work	29. Handle patient’s needs and problems immediately	3, 5
		30. Plan is sound and work is completed on time	4

* Subscales: 1, teachers; 2, therapists; 3, patients; 4, peer students; 5, learners.

**Table 6:** An example of MPC subscale: teacher subscales.

	Subscale: Teacher’s Checklist Items	Scores *				
		1	2	3	4	5
1	Communicate with patients and families in an easy-to-understand manner					
2	Demonstrate professional clinical reasoning					
3	Carefully assess symptoms and needs for assistive technology interventions					
4	Understand the skills and details of assistive technology service					
5	Understand the concept, evaluation process, and intervention methods of assistive technology					
6	Understand basic body structure and biomechanics related to diseases and diagnosis					
7	Ability and knowledge to develop organizational sequence for making assistive technology					
8	Provide easy-to-understand health education for patients (including usage, side effects, prognosis)					
9	Understanding the spirit of the AT service					
10	Innovation and creativity					
Subtotal Score =						

* 1 = very poor; 2 = poor; 3 = normal; 4 = good; 5 = excellent.

## Data Availability

All data generated or analyzed during this study are included in this published article.
